# Anesthetic and Analgesic Management of Pediatric Patients for Staged Repeat Same‐Day Surgery

**DOI:** 10.1155/cria/7047720

**Published:** 2026-04-06

**Authors:** Martha O. Herbst, Katherine G. C. Keech, Bryan S. Brindeiro, Kristen G. Berrebi, Jennifer G. Powers, Michelle N. Bremer Gama, Stephen R. Hays

**Affiliations:** ^1^ University of Iowa, Roy J. & Lucille A. Carver College of Medicine, Department of Anesthesia, Iowa City, Iowa, USA, uiowa.edu; ^2^ University of Iowa, Roy J. & Lucille A. Carver College of Medicine, Department of Pediatrics, Iowa City, Iowa, USA, uiowa.edu; ^3^ University of Iowa, Roy J. & Lucille A. Carver College of Medicine, Department of Dermatology, Iowa City, Iowa, USA, uiowa.edu

**Keywords:** dermatofibrosarcoma protuberans (DFSP), epidurography, Mohs micrographic surgery, pediatric analgesia, pediatric anesthesia, pediatric epidural, tunneled epidural catheter

## Abstract

**Background:**

Dermatofibrosarcoma protuberans (DFSP) is a rare dermatologic malignancy in children requiring a multidisciplinary approach for surgical excision and postoperative care. The modified “slow Mohs” repeat staged excision and the associated anesthetic technique are not well described in the pediatric literature.

**Case Presentation:**

We describe three pediatric patients whose staged excision procedures were completed using general anesthesia plus systemic multimodal analgesia (all patients), including tunneled epidural catheters (two patients with truncal tumors) or a scheduled long‐acting opioid (one patient with a forehead lesion). Complete dermatologic excision and excellent surgical analgesia were achieved in all patients. Specific interventions to verify epidural catheter position and allow potential prolonged catheter use were of particular utility in perioperative management.

**Conclusions:**

Children with DFSP present a unique challenge to provide optimal surgical and cosmetic results with serial staged Mohs excision while ensuring adequate ongoing analgesia. Close interdisciplinary communication and advance planning are essential. Multimodal analgesia, including tunneled epidural catheters if anatomically appropriate, or scheduled long‐acting opioid, is a key component of successful management. Confirmation of epidural catheter position likely improves analgesic efficacy and reduces the need for catheter replacement. We suggest that the care of pediatric patients undergoing slow Mohs staged repeat excision is best undertaken in a tertiary care setting with adequate multidisciplinary subspecialist support.

## 1. Introduction

Dermatofibrosarcoma protuberans (DFSP), a slow‐growing dermatologic sarcoma that is rare in children, may be managed with single‐session wide local excision or staged resection [1]. The latter has been recommended to maximize cosmesis and minimize the risk of recurrence [2]. For adult patients, serial Mohs surgery with local anesthesia is performed in the office or clinic in a single session. Such an approach in children is often impractical or impossible. A “slow Mohs” approach utilizing interval serial resections can be modified for pediatric patients. The modified slow Mohs micrographic surgical method utilized for our patients involved general anesthesia and surgery in the morning, followed by repeat excision under general anesthesia after pathology review in the afternoon, allowing for removal of large lesions while preserving as much healthy tissue as possible. Each patient’s first procedure took place in the morning, followed by pathologic review of the specimen to assess margins. If margins were positive, the patient returned to the OR later that day for additional resection. Staged resection in the morning and again in the afternoon continued for as many days as needed until negative margins were obtained, after which a final procedure for site closure was performed.

Sequential procedures potentially involving resection of large areas of skin necessitated optimal ongoing analgesia. We placed tunneled epidural catheters in two patients with truncal tumors. In one patient with a forehead lesion, we achieved excellent ongoing analgesia utilizing a multimodal analgesic regimen with a sustained‐release opioid. All patients achieved complete dermatologic excision with excellent pain control throughout.

## 2. Case Reports

Families of patients verbally agreed to report these cases. In accordance with institutional requirements, written consent for the use of patient photographs was obtained from families.

### 2.1. Case 1

**FIGURE 1:**
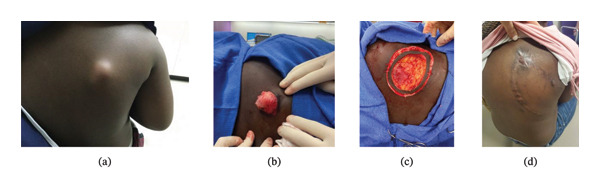
Case 1. (a) Preoperative mass. (b) Intraoperative mass. (c) Intraoperative excision. (d) Postoperative scar.

**FIGURE 2:**
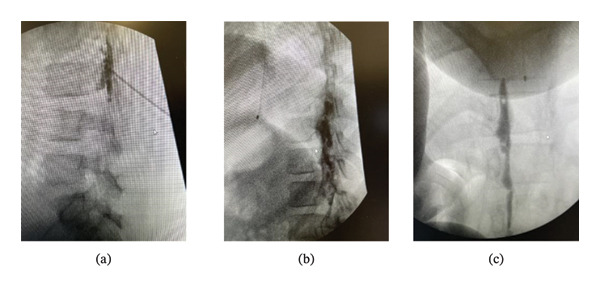
Intraoperative epidurograms. (a) Contrast via insertion needle at L3‐4, lateral view. (b) Contrast via epidural catheter with tip at T7, lateral view. (c) Contrast via epidural catheter with tip at T7, AP view. Abbreviations: AP: anteroposterior; L3‐4: third−fourth lumbar vertebral level; T7: seventh thoracic vertebral level.

An otherwise healthy 40 kg patient in middle childhood presented for staged Mohs excision of a DFSP over the right scapula (Figure 1(a)). For the initial procedure, the patient received general anesthesia with endotracheal intubation and controlled mechanical ventilation, propofol during induction, and sevoflurane maintenance without noted complications. A tunneled epidural catheter was placed under general anesthesia. Excisional boundaries were expected to cross the midline, preventing at‐level congruent catheter placement, so a fluoroscopically guided epidural was placed at the third−fourth lumbar interspace (L3‐4) and threaded cranially to the seventh thoracic vertebral level (T7). Fluoroscopic confirmation of insertion needle and catheter position was obtained (Figure 2), and the catheter was tunneled. Epidural infusion of 0.1% ropivacaine + hydromorphone 2.5 mcg/mL at 6 mL/h with nurse‐controlled epidural analgesia (NCEA) bolus dosing 3 mL every 30 min provided excellent analgesia. The patient also received scheduled intravenous (IV) ketorolac. No oral or IV opioid was required.

Staged excisions occurred on three separate occasions (Day 1 morning, Day 1 afternoon, and Day 2 morning) (Figures 1(b) and 1(c)), followed by closure (Day 2 PM) after demonstration of adequate surgical margins. Dermatopathology revealed fibrosarcomatous transformation. There was no evidence of pulmonary metastases on computed tomography (CT). The patient later developed a wound seroma and secondary infection requiring rehospitalization after initial discharge, ultimately healing well (Figure 1(d)). Given evidence of fibrosarcomatous transformation, follow‐up care will include evaluation in the dermatology clinic and surveillance chest CT scan every 3 months.

### 2.2. Case 2

An otherwise healthy 53 kg, early school‐age child presented for staged Mohs resection of a DFSP of the anterior chest wall. For the first procedure, a tunneled epidural catheter was placed at the fourth thoracic vertebral level (T4) after induction of general anesthesia with a supraglottic airway. Epidural infusion of 0.1% ropivacaine with hydromorphone 5 mcg/mL and clonidine 0.25 mcg/mL at 4 mL/h with NCEA bolus dosing of 2 mL every 30 min provided excellent analgesia. No other intraoperative opioid was given. Epidural hydromorphone concentration was decreased to 2.5 mcg/mL on the first postoperative day (POD 1) for pruritus, causing the patient to scratch at the face, with prompt improvement. With PCEA and scheduled oral acetaminophen and ibuprofen, the patient reported pain scores of 0/10 throughout. No oral or IV opioid was required.

**FIGURE 3:**
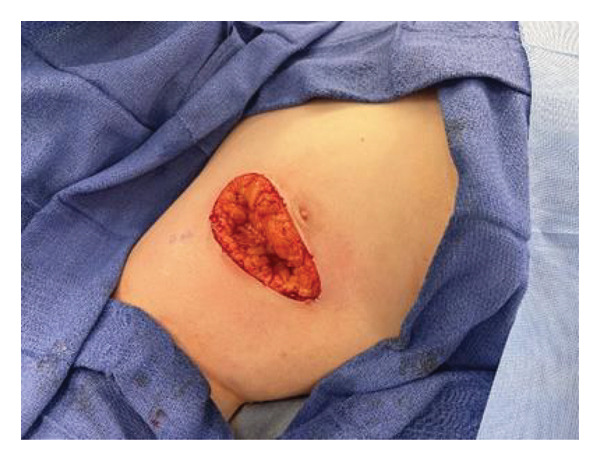
Case 2. Intraoperative excision.

After two staged Mohs resections, surgical margins were negative (Figure 3). Surgical closure was performed at the third procedure. Although dermatopathology indicated no evidence of fibrosarcomatous transformation, the tumor was < 0.1 cm from the inferior margin. Follow‐up care will include evaluation in the dermatology clinic and chest radiography every 6 months.

### 2.3. Case 3

A 55 kg, teenage patient presented for staged Mohs resection of an atrophic type DFSP on the forehead. The patient had a history of anxiety and obsessive‐compulsive disorders with prior inpatient psychiatric admissions, but tolerated an initial skin biopsy in the office. Preresection satellite biopsies of the glabella and superior central forehead were negative.

Due to the lesion location on the forehead, an indwelling catheter was felt to be impractical, so an alternative multimodal analgesic plan was developed in advance. The patient underwent three staged Mohs resections with general anesthesia on the first day (morning, early afternoon, and late afternoon), followed by frontal bone burring with biopsy and site closure with general anesthesia on the second day.

For the first procedure, the patient received IV acetaminophen 15 mg/kg, dexamethasone 0.1 mg/kg, and methadone 0.1 mg/kg, as well as IV fentanyl and ketamine at induction. For the second Mohs resection, approximately 3 h following the first, the patient received IV methadone 0.1 mg/kg and ketamine 0.5 mg/kg. For the third Mohs resection, approximately 4 h after the second, the patient again received IV methadone 0.1 mg/kg and ketamine 0.5 mg/kg.

The postoperative analgesic regimen included scheduled oral acetaminophen, scheduled oral methadone 5 mg every 12 h, and demand‐only hydromorphone IV patient‐controlled analgesia (PCA) 0.2 mg every 15 min. The patient had consistent pain scores of 0/10 and never used the PCA, which was discontinued on POD 1 on patient′s request. On POD 2, scheduled oral methadone was transitioned to oral oxycodone 5 mg every 3 h as needed. Pain scores increased to 4–6/10 but were acceptable to the patient. After discharge, the patient required 2 days of as‐needed oral oxycodone, with excellent subsequent analgesia on scheduled oral ibuprofen. Follow‐up in the dermatology clinic revealed excellent wound healing and cosmesis. Given adequate margins without fibrosarcomatous transformation, no specific follow‐up was recommended.

## 3. Discussion

DFSP is a rare malignancy generally diagnosed in middle‐aged adults, most often on the trunk [3], accounting for about 0.1% of all adult malignancies. The incidence in Black patients is approximately twice that in White patients. About 10% of DFSP occurs at a site of previous trauma. Pediatric patients have similar areas of DFSP involvement as adults, with truncal locations being most common, followed by upper extremities, lower extremities, and head and neck. Children account for about 8% of patients with DFSP, of which 15% are congenital. There is a slight female predominance in pediatric DFSP.

Staged excision of DFSP (“slow Mohs”) approach in children has been shown to be a safe and effective management [4]. Care of pediatric patients with DFSP is best undertaken in a tertiary care setting with multiple subspecialty care teams including pediatric dermatology, pediatric hematology−oncology, and pediatric anesthesiology with a pediatric pain service. Admission to pediatric hematology−oncology with other services consulting has worked well at our institution.

Repeated excisions of large areas of skin may be quite painful. Inadequate analgesia in children is associated with longer hospital stays, reduced patient and family satisfaction, and increased risk of morbidity and mortality [5]. Multimodal analgesic regimens utilizing various combinations of pain management strategies improve pain control, reduce child and parental anxiety, and decrease opioid consumption [6]. Combinations of multimodal analgesic regimens and regional anesthetic techniques may be particularly effective in reducing opioid requirement, with associated reduction in risk of opioid‐associated adverse effects [7].

Perioperative epidural analgesia offers numerous potential benefits. Epidural analgesia in children minimizes hemodynamic alterations, facilitates earlier recovery, and promotes rapid weaning from mechanical ventilation [8]. Epidural analgesia also reduces systemic opioid consumption, time spent in a catabolic state, circulating stress hormone levels, and length of hospitalization [9, 10]. Although usually placed with sedation or anesthesia, epidural analgesia in children is safe, with minimal, usually transient, complications [11].

In our patients, as in our usual practice, we subcutaneously tunneled the epidural catheters to reduce the risk of migration and infection and allow prolonged duration of epidural analgesia. Placement of tunneled epidural catheters allows catheters to remain in place for weeks to months [12] or longer. Catheter migration with a change in tip position can occur even with tunneled catheters and is more clinically significant in smaller patients [13]. In practice, clinically significant migration in older children and adults is uncommon.

Tunneling epidural catheters reduces colonization rate, with no correlation between the length of catheter use longer than 24 h and bacterial colonization [14]. Long‐term epidural catheter use may be particularly useful in patients with prolonged severe pain associated with extensive surgery, trauma, vascular insufficiency, or sickle cell disease, and for palliative analgesia. We tunneled the epidural catheters in our patients, given the uncertainty regarding the duration of serial staged Mohs excision.

Use of imaging during epidural catheter placement in adults allows for targeted placement, improving reliability, analgesic efficacy, and likely overall safety [15]. Various imaging modalities, including ultrasound [16], have been recommended to verify epidural catheter position at the time of placement in children. Pediatric epidural catheters are commonly placed with sedation or general anesthesia, limiting the ability to assess catheter efficacy. Similarly, when catheters are placed or replaced postoperatively, sedation or anesthesia is commonly required. We favor imaging confirmation of epidural catheter position at the time of placement to increase the likelihood of adequate analgesia and minimize the need for catheter replacement.

## 4. Conclusions

Children with DFSP present a unique challenge to provide optimal surgical and cosmetic results with adequate ongoing analgesia. Close interdisciplinary communication and advance planning are essential. Our case series suggests multimodal analgesia, including tunneled epidural catheters if anatomically appropriate, or scheduled long‐acting opioid, is a key component of successful management. Confirmation of epidural catheter position at the time of placement likely improves analgesic efficacy and reduces the need for catheter replacement. Care of pediatric patients undergoing slow Mohs staged repeat excision is best undertaken in a tertiary care setting with adequate multidisciplinary subspecialist support.

**Nomenclature**
AP: Anteroposterior
CT: Computed tomography
DFSP: Dermatofibrosarcoma protuberans
IV: Intravenous
NCEA: Nurse‐controlled epidural analgesia
NSAIDs: Nonsteroidal anti‐inflammatory drugs
PCA: Patient‐controlled analgesia
PCEA: Patient‐controlled epidural analgesia
POD: Postoperative day
T, L: Thoracic, lumbar vertebral level (followed by appropriate numbers)

## Author Contributions

Martha O. Herbst, MD, designed perioperative pain plans and implemented them for the first and third patients and wrote the majority of the manuscript and served as primary manuscript editor.

Katherine G. C. Keech helped write the discussion and edit the manuscript.

Bryan S. Brindeiro helped write the discussion and edit the manuscript.

Kristen G. Berrebi was the proceduralist for all patients, helped write sections pertinent to dermatologic management, and helped edit the manuscript.

Jennifer G. Powers helped write sections pertinent to dermatologic management and helped edit the manuscript.

Michelle N. Bremer Gama helped write the discussion.

Stephen R. Hays primarily developed perioperative anesthetic and analgesic plans for patients, provided guidance for manuscript development, and edited the final manuscript.

## Funding

The authors have no sources of funding to declare for this manuscript.

## Conflicts of Interest

The authors declare no conflicts of interest.

## Data Availability

Data sharing is not applicable to this article as no datasets were generated or analyzed during the current study.
